# Clinical and Psychosocial Outcomes Associated With a Tele-behavioral Health Platform for Families: Retrospective Study

**DOI:** 10.2196/43600

**Published:** 2023-03-17

**Authors:** Theoren Loo, Justin Hunt, David Grodberg, Dena Bravata

**Affiliations:** 1 Brightline Palo Alto, CA United States; 2 Child Study Center Yale University School of Medicine New Haven, CT United States; 3 Center for Primary Care and Outcomes Research Stanford Health Policy, Freeman Spogli Institute and Stanford School of Medicine Stanford University Palo Alto, CA United States

**Keywords:** adolescent, child, family health, resilience, psychological, mental health, caregiver stress, anxiety, depression, behavior, program, educational psychotherapy, psychiatry, care

## Abstract

**Background:**

The burden of pediatric mental illness in the United States has steadily worsened over the past decade. A recent increase in employer-sponsored behavioral health programs has focused on the needs of the general population. However, these programs do not provide the specialty mental health care required for children, adolescents, and their families.

**Objective:**

This study aimed to evaluate the effects of a technology-enabled pediatric and family behavioral health service on clinical outcomes among children and caregiver strain. The service is available to commercially insured populations and provides educational content; tele-behavioral health care, including coaching, therapy, and psychiatry; and care escalation and coordination.

**Methods:**

A retrospective cohort analysis of members using the service between February and September 2022 was conducted. Clinical outcomes for children and their caregivers were collected using the Pediatric Symptom Checklist-17 (PSC-17), Generalized Anxiety Disorder 7-item (GAD-7), Patient Health Questionnaire 8-item (PHQ-8), and Caregiver Strain Questionnaire-Short Form 7 (CGSQ-SF7). Rates of reliable improvement were determined by calculating the reliable change index for each outcome. Paired, 2-tailed *t* tests were used to evaluate significant changes in assessment scores at follow-up compared to baseline.

**Results:**

Of the 4139 participants who enrolled with the service, 48 (1.2%) were referred out for more intensive care, 2393 (57.8%) were referred to coaching, and 1698 (41%) were referred to therapy and psychiatry. Among the 703 members who completed the intervention and provided pre- and postintervention outcomes data, 386 (54.9%) used psychoeducational content, 345 (49.1%) received coaching, and 358 (50.9%) received therapy and psychiatry. In coaching, 75% (183/244) of participants showed reliable improvement on the PSC-17 total score, 72.5% (177/244) on the PSC-17 internalizing score, and 31.5% (105/333) on the CGSQ-SF7 total score (average improvement: PSC-17 total score, 3.37 points; *P*<.001; PSC-17 internalizing score, 1.58 points; *P*<.001; and CGSQ-SF7 total score, 1.02 points; *P*<.001). In therapy and psychiatry, 68.8% (232/337) of participants showed reliable improvement on the PSC-17 total score, 70.6% (238/337) on the PSC-17 internalizing score, 65.2% (219/336) on the CGSQ-SF7 total score, 70.7% (82/116) on the GAD-7 score, and 67.5% (77/114) on the PHQ-8 score (average improvement: PSC-17 total score, 3.16 points; *P*<.001; PSC-17 internalizing score, 1.66 points; *P*<.001; CGSQ-SF7 total score, 1.06 points; *P*<.001; GAD-7 score, 3.00 points; *P*<.001; and PHQ-8 score, 2.91 points; *P*<.001).

**Conclusions:**

Tele-behavioral health offerings can be effective in improving caregiver strain and psychosocial functioning and depression and anxiety symptoms in a pediatric population. Moreover, these digital mental health offerings may provide a scalable solution to children and their families who lack access to essential pediatric mental health services.

## Introduction

The burden of pediatric mental illness in the United States has steadily worsened over the past decade [1,2]. Prior to the pandemic, 1 in 6 (17.4%) children aged 2 to 8 years in the United States was diagnosed with a mental health condition [3], and among adolescents aged 12 to 17 years, 36.7% had persistent feelings of sadness or hopelessness and 8.9% attempted suicide [4]. More recent data suggest that the COVID-19 pandemic has dramatically exacerbated these trends, with 22% of children aged 3 to 17 years currently affected by mental, emotional, developmental, or behavioral conditions [5]. Unfortunately, currently only about 20% of them receive care from a specialized provider [5], and 62% of children with persistent mental health symptoms have not had any care from a pediatric mental health provider [6]. This low use of mental health services is even more worrisome considering the recent increase in rates of teen suicide (eg, a doubling in the rate of emergency department visits for self-harm between 2001 and 2019) [7].

Delayed treatment of pediatric behavioral health issues can have long-lasting sequelae given that half of all lifetime mental health disorders have a childhood or adolescent onset [8]. Although poor access to pediatric care is often associated with a lack of knowledge about child mental health, lack of knowledge or access to help, and stigmatization of mental health disorders [9], workforce shortages have acutely exacerbated the already long waitlists for pediatric mental health care providers, leading to delayed intervention. In response to the enormous gap between pediatric behavioral health needs and access to care, the American Academy of Pediatrics, American Academy of Child and Adolescent Psychiatry, and Children’s Hospital Association have declared a pediatric mental health crisis and called for effective and sustainable models of pediatric mental health care to address the needs of children with behavioral health concerns [10]. Relatedly, in May 2021, the US Department of Health and Human Services announced a US $14.2 million rescue plan to expand access to pediatric behavioral health services [5].

Among commercially insured populations, the costs for pediatric outpatient behavioral care increased by 84% between 2018 and 2021 compared to an increase of 60% in behavioral health costs for the general population during that same interval [2]. Self-insured employers pay a disproportionate share of these costs because they provide direct payment for mental health services and bear indirect costs associated with parental productivity losses. In response, 78% of self-insured employers have implemented behavioral health benefits, including employee assistance programs and others that increase access for families to lower cost-sharing mental health services such as tele-mental health [2]. However, the preponderance of these programs is for the general population, largely designed for the care of adults, and do not specialize in the needs of children and their families. Moreover, children and teens have largely been left out of recent telehealth expansion for behavioral health services [11]. Pediatric mental health services require a highly specialized approach that tailors interventions to match a child’s developmental level; furthermore, care must include parents in shared decision-making; informed consent processes; and skills-based, parent-mediated interventions such as parent management training (PMT) for disruptive behaviors or suspected attention-deficit/hyperactivity disorder in young children [12-15].

In response to these trends, we implemented a comprehensive, technology-enabled pediatric and family behavioral health service for commercially insured populations. The intervention provides access to educational content; tele-behavioral health care, including coaching, therapy, and psychiatry; and care escalation and coordination. Patients and their families receive either coaching or therapy and psychiatry based on their clinical acuity and clinical progress on validated metrics. For children with acute care needs, the service provides care escalation and referral to higher levels of care (eg, emergency departments, intensive outpatient programs, and inpatient units) as clinically indicated. The aim of this paper was to present a retrospective cohort analysis of the clinical outcomes associated with this service. We anticipate that these results will inform the ongoing development of scalable, employer-sponsored tele-behavioral health offerings for children and their families.

## Methods

### Intervention

Brightline is a technology-enabled behavioral health care service for children (aged 18 months to 17 years) and their families provided to commercially insured populations in all 50 states through their employer and health plan benefits [16]. The intervention has 4 main components: psychoeducational content, coaching, therapy and psychiatry, and care escalation and coordination.

#### Psychoeducational Content

All members have access to a rich library of educational content addressing common behavioral health challenges for children and their families. Discrete, interactive, and evidence-informed content packages (eg, videos, articles, and exercises) on specific topics (eg, parent behavior training, problem-solving, and relaxation techniques) can be used alone or as a supplement to coaching and clinical care. Content includes long- and short-form modules (consumable in 1 to 10 minutes) and is audience specific (modules are designed specifically to meet age-appropriate concerns; some are intended for children, whereas others are intended for teens, caregivers, and siblings). The materials have been extensively tested and revised based on member feedback.

#### Tele-behavioral Health Coaching

All members also have access to behavioral health coaches via asynchronous web-based chat messaging and to protocolized, evidence-informed, and one-to-one synchronous coaching. The coaching programs are preventive interventions for those with subclinical needs designed to help members build specific skills (eg, PMT, organizational skills, sleep hygiene, stress reduction, problem-solving, and social communication). Coaching programs typically last 4 to 6 weeks, are highly protocolized, and are implemented via a live telehealth platform with the same coach. The programs are designed to be either parent- or child-focused. For example, the standard-of-care approach involves an assessment of the child that includes meeting with parents to understand their concerns, taking the child’s developmental history, and performing an observational assessment. After a diagnostic formulation is completed and PMT is recommended, we begin by providing basic psychoeducation to the parents that is followed by a skills-based intervention. PMT skills are taught using an incremental approach and include domains such as (1) understanding how to identify positive behaviors to focus on (as opposed to focusing on the child’s negative behaviors); (2) understanding and demonstrating effectiveness in using praise and other types of rewards to reinforce the child’s positive behavior; and (3) setting up behavioral plans and troubleshooting their implementation [17]. Alternatively, a school-age child or teen with subclinical worries may benefit from learning cognitive behavioral therapy–informed skills such as using a “fear thermometer” to track their worries [18]. Coaches choose from an extensive library of digital interventions to support members during their daily routines to promote the practice of new skills as well as to provide help overcoming barriers.

Coaches are non-licensed providers who have either a (1) master’s degree in psychology or a related field plus at least 1 year of experience in behavioral health including child- and family-focused cognitive behavioral therapy; motivational interviewing; and Specific, Measurable, Assignable, Realistic, and Time–related goal setting [19] or (2) bachelor’s degree in a health-related field and have National Board Certified Health & Wellness Coach or International Coaching Federation certification, plus 5 or more years of relevant experience including direct service in a behavioral health setting working with children or families. Coaches receive training on behavioral change theory, motivational strategies, and health education and promotion theories, which help parents and children develop intrinsic motivation and obtain skills to create sustainable change for improved health and well-being. The coaches’ work is highly structured and protocol bound, and a quality assurance system allows for continual oversight of quality and fidelity to the model.

#### Therapy and Psychiatry

Children and teens who require a higher level of clinical support are referred to an internal licensed therapist for evidence-based, family-centric psychotherapy via the Modular Approach to Therapy for Anxiety, Depression, Trauma, or Conduct Problems model [20]. Additionally, referrals to the internal psychiatry team for medication management are also available. Since only 3 children required psychiatry and all of them also received therapy during the study interval, their outcomes are considered part of the therapy and psychiatry intervention.

#### Care Escalation and Coordination

The service provides external referral for immediate treatment and related care coordination for children or teens who indicate plans to harm themselves or others or have required admission to a psychiatric hospital or residential treatment facility within the previous 30 days, complicating substance use, or severely disordered eating. Additionally, for children and caregivers who disclosed clinical risk in the context of a coaching session, a case review protocol involving the coach and a manager ensured prompt escalation to clinical services. Typically, these referrals include a warm handoff to an external intake team, closing the loop with the family and other stakeholders, and the maintenance of safety planning. Even when members no longer require a higher level of care, they continue to receive behavioral health care coordination.

### Participants

Members who enrolled during the 9-month interval between February and September 2022 were included in this study. Participants must have completed at least one coaching or therapy and psychiatry session and completed a baseline and follow-up assessment to be included in this analysis.

### Data Collected

Participants completed validated, self-reported assessments to evaluate the clinical effectiveness of the intervention for both the children and their parents. Baseline assessments were collected on the web before engagement with coaches and clinicians, and follow-up assessments were administered every 4 weeks.

We collected data using the Pediatric Symptom Checklist-17 (PSC-17) and Caregiver Strain Questionnaire-Short Form 7 (CGSQ-SF7) from all participants receiving tele-behavioral health coaching. The PSC-17 is a parent-completed survey that measures a child’s total psychosocial functioning, with subscales that measure function in the areas of internalizing, attention, and externalizing problems [21]. Because the coaching intervention was designed primarily to treat internalizing behaviors, the PSC-17 total score and internalizing scores were the outcomes of focus for this analysis. The CGSQ-SF7 is a parent-completed assessment of the impact of a child’s emotional and behavioral problems on caregivers [22].

Given the higher acuity of participants receiving therapy and psychiatry, in addition to the PSC-17 and CGSQ-SF7, teen participants also completed the Generalized Anxiety Disorder 7-item (GAD-7) [23] to assess symptom severity for generalized anxiety disorder and the Patient Health Questionnaire 8-item (PHQ-8), which is the 9-item PHQ without the last question regarding suicidality, to measure the severity of depressive disorders [24]. Question 9 of the 9-item PHQ was asked during the member’s tele-behavioral health session to ensure appropriate follow-up.

### Analyses

This retrospective cohort study evaluated the effects of tele-behavioral health care, including coaching and therapy using baseline and follow-up responses. Counts of participants with reliable improvement and reliable deterioration across all assessments were determined using the reliable change index (RCI) [25]. The RCI scores were computed per Jacobson and Truax [26], where an RCI score of 1.96 or higher indicates improvement and a score of –1.96 or lower indicates deterioration [20]. Paired, 2-tailed *t* tests were used to evaluate significant changes in assessment scores from baseline. Bonferroni correction was used to adjust the α level to control for multiple comparisons. Cohen *d* was also used to measure the effect size of coaching and therapy across all assessments. All statistical analyses were performed in R software (version 4.1.2; R Foundation for Statistical Computing).

### Ethical Considerations

This study was classified as exempt from consent requirements under human subjects review and approved by WIRB-Copernicus Group IRB (protocol: Brightline.002, February 17, 2022). Study data were deidentified and stored on a Health Insurance Portability and Accountability Act–compliant hosting platform using industry standard encryption. In addition, host-based intrusion detection and routine vulnerability scans ensure that the data are secure at all times. The intervention was provided to participants by their employer or insurance plan. Participants received no additional compensation for participation.

## Results

### Participants

**Figure 1:**
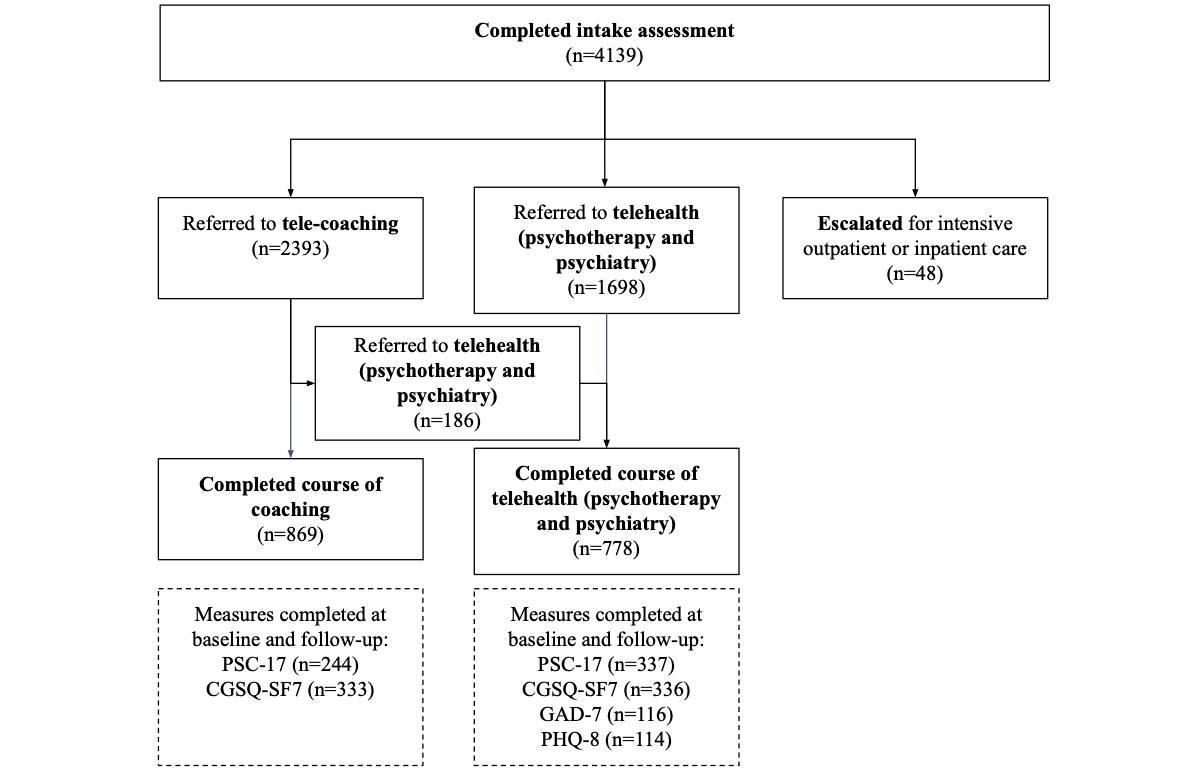
Participant flow through interventions. CGSQ-SF7: Caregiver Strain Questionnaire-Short Form; GAD-7: Generalized Anxiety Disorder 7-Item; PHQ-8: Patient Health Questionnaire 8-Item; PSC-17: Pediatric Symptom Checklist-17.

Of the 4139 participants who created an account, 48 (1.2%) were referred out for more intensive care, 2393 (57.8%) were referred to coaching, and 1698 (41%) were referred to therapy and psychiatry. Of those referred, 869 (36.3%) completed a course of coaching and 778 (45.8%) completed a course in therapy and psychiatry (Figure 1).

This study included 703 participants who completed a baseline and follow-up assessment, with 345 (49.1%) enrolled in coaching and 358 (50.9%) enrolled in therapy and psychiatry (Table 1). Because the 48 patients referred for escalated intensive outpatient or inpatient care did not provide completed postintervention assessments, they were excluded from this analysis.

The majority (n=414, 58.9%) of participants were White. More male children received coaching (179/345, 51.9%) than therapy (141/358, 39.4%; *P*<.001), and the average age of children who received coaching was 10.0 years compared to 11.9 years for therapy and psychiatry (*P*<.001; Table 1). Among those referred for therapy and psychiatry, 44.4% (159/358) were teenagers.

**Table 1:** Demographic characteristics of study participants.

Demographics		Total participants (n=703), n (%)	Participants in coaching (n=345), n (%)	Participants in therapy and psychiatry (n=358), n (%)	*P* value	
**Race**						.47
	Asian	60 (8.5)	34 (9.9)	26 (7.3)		
	Black or African American	39 (5.5)	16 (4.6)	23 (6.4)		
	Hispanic	70 (10)	31 (9)	39 (10.9)		
	Multiracial	82 (11.7)	44 (12.8)	38 (10.6)		
	Other	16 (2.3)	9 (2.6)	7 (2)		
	Prefer not to say	22 (3.1)	10 (2.9)	12 (3.4)		
	White	414 (58.9)	201 (58.3)	213 (59.5)		
**Sex**						<.001
	Female	382 (54.3)	166 (48.1)	216 (60.3)		
	Male	320 (45.5)	179 (51.9)	141 (39.4)		
	Missing value	1 (0.1)	0 (0)	1 (0.3)		
**Age at enrollment (years)**						<.001
	≥5	47 (6.7)	45 (13.0)	2 (0.6)		
	6-9	196 (27.9)	103 (29.9)	93 (26)		
	10-12	203 (28.9)	99 (28.7)	104 (29.1)		
	13-17	257 (36.6)	98 (28.4)	159 (44.4)		

### Psychoeducational Content

All members had access to web-based educational content. Out of all 703 members, 386 (54.9%) used the psychoeducational content. Of these 386 members, 208 (53.9%) received coaching and 178 (46.1%) received therapy and psychiatry. On average, members viewed the educational content for 2.2 (SD 1.80) days.

### Tele-behavioral Health Coaching

#### Use of Asynchronous Chat Service With Coaches

In addition to synchronous visits with coaches, therapists, and psychiatrists, all members had access to behavioral health coaches via synchronous chat. Among the 345 members who received coaching, 332 (96.2%) used the coach chat service, for an average of 6.0 (SD 4.99) days, and among the 358 members who received therapy and psychiatry, 315 (88%) used the coach chat service, for an average of 4.7 (SD 4.15) days. The coach chat service was typically used by caregivers to check in with their Brightline care team to coordinate, plan, and receive encouragement and support from their family’s coach.

#### PSC-17 Total and Internalizing Scores

Of the 244 participants in coaching who completed a PSC-17 assessment, 183 (75%) showed improvement and 35 (14.3%) showed deterioration in the PSC-17 total score (Table 2). PSC-17 total scores decreased from baseline (mean 13.56, SD 5.27) to follow-up (mean 10.19, SD 5.24), for an average improvement of 3.37 points (*t*_243_=12.51; *P*<.001). Cohen *d*=0.64 suggests that coaching has a medium treatment effect size on the PSC-17 total score (Table 3).

For internalizing subscale scores, 177 (72.5%) participants showed improvement and 29 (11.9%) showed deterioration (Table 2). PSC-17 internalizing scores decreased from baseline (mean 4.95, SD 2.26) to follow-up (mean 3.37, SD 1.86), for an average improvement of 1.58 points (*t*_243_=13.07; *P*<.001). Cohen *d=*0.75 suggests that coaching has a medium treatment effect size on the PSC-17 internalizing score (Table 3).

**Table 2:** Rates of reliable improvement and deterioration on the Pediatric Symptom Checklist-17 (PSC-17) and Caregiver Strain Questionnaire-Short Form 7 (CGSQ-SF7) among participants in tele-behavioral health coaching.

Reliable improvement or deterioration	PSC-17 total score^a^ (n=244), n (%)	PSC-17 internalizing score^b^ (n=244), n (%)	CGSQ-SF7 total score^c^ (n=333), n (%)
Reliable improvement	183 (75)	177 (72.5)	105 (31.5)
Reliable deterioration	35 (14.3)	29 (11.9)	6 (1.8)

^a^PSC-17 total score measures a child’s overall psychosocial functioning.

^b^PSC-17 internalizing score measures a child’s risk of depression and anxiety.

^c^CGSQ-SF7 total score measures the impact of children’s emotional and behavioral problems on caregiver stress.

**Table 3:** Pediatric Symptom Checklist-17 (PSC-17) and Caregiver Strain Questionnaire-Short Form 7 (GCSQ-SF7) scores among participants in tele-behavioral health coaching at baseline and follow-up.

Assessment	Baseline, mean (SD)	Follow-up, mean (SD)	Paired difference mean (95% CI)	*t* test (*df*)	*P* value^a^	Cohen *d*
PSC-17 total score^b^	13.56 (5.27)	10.19 (5.24)	3.37 (2.84-3.90)	12.51 (243)	<.001	0.64
PSC-17 internalizing score^c^	4.95 (2.26)	3.37 (1.86)	1.58 (1.34-1.82)	13.07 (243)	<.001	0.75
CGSQ-SF7 total score^d^	5.11 (1.71)	4.09 (1.43)	1.02 (0.86-1.18)	12.60 (332)	<.001	0.64

^a^*t* test *P* value is significant at .017 after adjusting for multiple comparisons using Bonferroni correction.

^b^PSC-17 total score measures a child’s overall psychosocial functioning.

^c^PSC-17 internalizing score measures a child’s risk of depression and anxiety.

^d^CGSQ-SF7 total score measures the impact of children’s emotional and behavioral problems on caregiver stress.

#### CGSQ-SF7 Total Score

Of the 333 participants in coaching with a CGSQ-SF7 assessment, 105 (31.5%) showed improvement and 6 (1.8%) showed deterioration in the CGSQ-SF7 total score (Table 2). CGSQ-SF7 total scores decreased from baseline (mean 5.11, SD 1.71) to follow-up (mean 4.09, SD 1.43), for an average improvement of 1.02 points (*t*_332_=12.60; *P*<.001). Cohen *d*=0.64 suggests that coaching has a medium treatment effect size on the CGSQ-SF7 total score (Table 3).

### Therapy and Psychiatry Outcomes

#### PSC-17 Total and Internalizing Scores

Of the 337 participants in therapy and psychiatry with a PSC-17 assessment, 232 (68.8%) showed improvement and 81 (24%) showed deterioration in the PSC-17 total score (Table 4). PSC-17 total scores decreased from baseline (mean 15.04, SD 5.65) to follow-up (mean 11.88, SD 6.54), for an average improvement of 3.16 points (*t*_336_=11.33; *P*<.001). Cohen *d*=0.52 suggests that therapy and psychiatry have a medium treatment effect size on the PSC-17 total score (Table 5).

For internalizing subscale scores, 238 (70.6%) participants showed improvement and 57 (16.9%) showed deterioration (Table 4). PSC-17 internalizing scores decreased from baseline (mean 5.88, SD 2.22) to follow-up (mean 4.22, SD 2.40), for an average improvement of 1.66 points (*t*_336_=12.92; *P*<.001). Cohen *d*=0.72 suggests that therapy and psychiatry have a medium treatment effect size on the PSC-17 internalizing score (Table 5).

**Table 4:** Rates of reliable improvement and deterioration across assessments collected from participants in therapy and psychiatry.

Reliable improvement or deterioration	PSC-17^a^ total score^b^ (n=337), n (%)	PSC-17 internalizing score^c^ (n=337), n (%)	CGSQ-SF7^d^ total score (n=336), n (%)	GAD-7^e^ score (n=116), n (%)	PHQ-8^f^ score (n=114), n (%)
Reliable improvement	232 (68.8)	238 (70.6)	219 (65.2)	82 (70.7)	77 (67.5)
Reliable deterioration	81 (24)	57 (16.9)	44 (13.1)	26 (22.4)	27 (23.7)

^a^PSC-17: Pediatric Symptom Checklist-17.

^b^PSC-17 total score measures a child’s overall psychosocial functioning.

^c^PSC-17 internalizing score measures a child’s risk of depression and anxiety.

^d^CGSQ-SF7: Caregiver Strain Questionnaire-Short Form 7; CGSQ-SF7 total score measures the impact of children’s emotional and behavioral problems on caregiver stress.

^e^GAD-7: Generalized Anxiety Disorder 7-item; GAD-7 score measures the severity of anxiety symptoms.

^f^PHQ-8: Patient Health Questionnaire 8-item; PHQ-8 score measures the severity of depressive symptoms.

**Table 5:** Assessment scores among participants in therapy and psychiatry at baseline and follow-up.

Assessment	Baseline, mean (SD)	Follow-up, mean (SD)	Paired difference mean (95% CI)	*t* test (*df*)	*P* value^a^	Cohen *d*
PSC-17^b^ total score^c^	15.04 (5.65)	11.88 (6.54)	3.16 (2.62-3.71)	11.33 (336)	<.001	0.52
PSC-17 internalizing score^d^	5.88 (2.22)	4.22 (2.40)	1.66 (1.40-1.91)	12.92 (336)	<.001	0.72
CGSQ-SF7^e^ total score	5.64 (1.68)	4.58 (1.82)	1.06 (0.89-1.23)	12.48 (335)	<.001	0.60
GAD-7^f^ score	10.16 (5.39)	7.19 (4.84)	3.00 (2.03-3.90)	6.27 (115)	<.001	0.58
PHQ-8^g^ score	10.68 (6.48)	7.76 (6.06)	2.91 (1.95-3.88)	6.00 (113)	<.001	0.46

^a^*t* test *P* value is significant at .01 after adjusting for multiple comparisons using Bonferroni correction.

^b^PSC-17: Pediatric Symptom Checklist-17

^c^PSC-17 total score measures a child’s overall psychosocial functioning.

^d^PSC-17 internalizing score measures a child’s risk of depression and anxiety.

^e^CGSQ-SF7: Caregiver Strain Questionnaire-Short Form 7; CGSQ-SF7 total score measures the impact of children’s emotional and behavioral problems on caregiver stress.

^f^GAD-7: Generalized Anxiety Disorder 7-item; GAD-7 score measures the severity of anxiety symptoms.

^g^PHQ-8: Patient Health Questionnaire 8-item; PHQ-8 score measures the severity of depressive symptoms.

#### CGSQ-SF7 Total Score

Of the 336 participants in therapy and psychiatry with a CGSQ-SF7 assessment, 219 (65.2%) showed improvement and 44 (13.1%) showed deterioration in the CGSQ-SF7 total score (Table 4). CGSQ-SF7 total scores decreased from baseline (mean 5.64, SD 1.68) to follow-up (mean 4.58, SD 1.82), for an average improvement of 1.06 points (*t*_335_=12.48; *P*<.001). Cohen *d*=0.60 suggests that therapy and psychiatry have a medium treatment effect size on the CGSQ-SF7 total score (Table 5).

#### GAD-7 Score

Of the 116 participants in therapy and psychiatry with a GAD-7 assessment, 82 (70.7%) showed improvement and 26 (22.4%) showed deterioration in the GAD-7 score (Table 4). GAD-7 scores decreased from baseline (mean 10.16, SD 5.39) to follow-up (mean 7.19, SD 4.84), for an average improvement of 3.00 points (*t*_115_=6.27; *P*<.001). Cohen *d*=0.58 suggests that therapy and psychiatry have a median treatment effect size on the GAD-7score (Table 5).

#### PHQ-8 Score

Of the 114 participants in therapy and psychiatry with a PHQ-8 assessment, 77 (67.5%) showed improvement and 27 (23.7%) showed deterioration in the PHQ-8 score (Table 4). PHQ-8 scores decreased from baseline (mean 10.68, SD 6.48) to follow-up (mean 7.76, SD 6.06), for an average improvement of 2.91 points (*t*_113_=6.00; *P*<.001). Cohen *d*=0.46 suggests that therapy and psychiatry have a small treatment effect size on the PHQ-8 score (Table 5).

## Discussion

Despite the growing literature on the use of digital mental health interventions for adults [27-29], few studies have reported on clinical outcomes from digital behavioral health interventions for children, teens, and their families [30]. This analysis contributes 3 key findings to this literature. First, children and teens receiving tele-behavioral health coaching and clinical care improved significantly in terms of their global behavioral health symptoms and internalizing behaviors. The stronger results on the internalizing subscale compared to the total PSC-17 score align with the current structure of our treatment model, which has more robust coaching and clinical services for participants with internalizing conditions such as depression and anxiety than for those with inattention or disruptive, externalizing presentations. An extension of the current intervention might include modalities that address externalizing behaviors such as PMT for disruptive and oppositional behavior.

Second, children and teens requiring therapy and psychiatry had significant improvements in their depression and anxiety. Coupled with the fact that 1% of the eligible population required referral to acute care providers, this finding suggests that digital mental health offerings for older children or teens and their parents can provide clinically meaningful support even among higher-acuity populations—an important finding in light of the poor access to timely, in-person care in many communities. The magnitude of improvement in depression and anxiety is similar to that seen in in-person studies [31,32].

Third, caregivers (most often parents) reported significantly reduced stress associated with the care of children with behavioral health needs. This was true across all levels of care. This finding is an important one for commercially insured populations, especially for self-insured employers interested in programs that can be deployed at scale for employees with affected family members. Future studies of such interventions should directly measure both caregiver stress and absenteeism and presenteeism to inform investment decisions.

Taken together, these findings support the concept that a digital mental health offering can support the needs of a clinically diverse, commercially insured pediatric population. This digital approach allows for measurement-based, multitiered care via initial triage and subsequent task shifting to coaching for children and adolescents with mild-to-moderate symptoms, thus opening up access to licensed clinicians for more complex presentations. Our findings corroborate previous research using similar digital, multitiered models in adult populations [27-29].

Our analysis has 4 key limitations. First, our retrospective observational design lacked a control group, preventing us from making any causal inferences. Second, the population studied was mostly White and may not be representative of all commercially insured populations. It will be important to oversample populations of diverse socioeconomic backgrounds in the future. Third, given the small number of children requiring medication management, we were unable to report their outcomes separately. This will be key to understanding the extent to which a digital offering, such as the intervention studied here, can treat children with conditions (eg, attention-deficit/hyperactivity disorder) that require pharmacological interventions. Finally, given the short duration of follow-up, we do not have long-term follow-up data for either the children referred for acute care follow-up or for those in the core intervention.

This analysis of a digital, multitiered pediatric mental health treatment model demonstrates positive impacts on mental health outcomes per the widely used and validated psychometric measures for both children and their parents. We believe this study and planned future work will inform the ongoing development of digital pediatric mental health models for commercially insured populations.

## Abbreviations

CGSQ-SF7: Caregiver Strain Questionnaire-Short Form 7
GAD-7: Generalized Anxiety Disorder 7-item
PHQ-8: Patient Health Questionnaire 8-item
PMT: parent management training
PSC-17: Pediatric Symptom Checklist-17
RCI: reliable change index

## Data Availability

The data sets generated and analyzed during the current study are available from the corresponding author on reasonable request.
